# A diagnostic model for NTM disease in HIV-positive patients: a machine learning-based analysis with novel inflammatory markers

**DOI:** 10.3389/fimmu.2025.1652747

**Published:** 2025-11-14

**Authors:** Longfen Li, Chunjing Shi, Xing Liu, Wenming Li, Yun Luo, Huajie Zhang, Ge Wang, Yanhong Zhao, Yuanqing Huang, Juan Yang, Jiao Yang, Shengan Li, Lingjun Shen

**Affiliations:** 1 Tuberculosis Department 3, The Third People's Hospital of Kunming, Kunming, China; 2 Tuberculosis Department 3, Yunnan Provincial Clinical Medical Center for Infectious Diseases, Kunming, China; 3 Tuberculosis Department 3, Medical Technology Center for the Diagnosis and Treatment of Non-tuberculous Mycobacterial Disease, Kunming, China; 4 Department of Pharmacy, The Third People's Hospital of Kunming, Kunming, China; 5 Department of Pathogen Biology and Immunology, Faculty of Basic Medical Science, Kunming Medical University, Kunming, China

**Keywords:** machine learning, HIV, non-tuberculous mycobacterial disease, new inflammatory biomarkers, diagnostic model, SHapley Additive exPlanations

## Abstract

**Background:**

Globally, the prevalence of nontuberculous mycobacterial (NTM) co-infection among HIV-positive patients is increasing. The diagnosis of HIV-positive patients co-infected with NTM relies on mycobacterial culture and identification, as well as molecular biology techniques. However, culture-based methods are technically challenging, time-consuming, and costly. Therefore, it is urgent to explore early diagnostic methods for HIV-positive patients co-infected with NTM. To address this issue, the present study had aimed to explore new approaches for the early diagnosis of NTM disease in HIV-positive patients. This study aimed to thoroughly investigate the potential value of novel inflammatory markers in the early diagnosis of nontuberculous mycobacterial (NTM) disease among HIV-positive patients using machine learning techniques, thereby providing a scientifically sound and clinically feasible diagnostic basis for the early identification of this condition in clinical practice.

**Methods:**

A retrospective analysis was conducted on 4,496 HIV-infected individuals admitted to the Third People’s Hospital of Kunming, between August 1, 2021, to August 31, 2024.Based on the inclusion and exclusion criteria, a total of 78 HIV-infected individuals with NTM disease were finally included as the experimental group, and 187 HIV-positive patients without NTM disease were included as the control group. Clinical data of the participants were collected. For comparisons between groups, the chi-square (χ^2^) test or the nonparametric Mann-Whitney U test was used as appropriate. Indicators with *p* < 0.05 in the comparison between the two groups were subjected to LASSO regression for variable screening. Subsequently, Logistic regression(LR), Random Forest(RF), and Support Vector Machine-Recursive Feature Elimination (SVM-RFE) were employed for further variable selection. To assess multicollinearity among variables, tolerance and variance inflation factor (VIF) were used as criteria. LR, RF, and SVM models were established. All the subjects included in the study were assigned to the training set, and 3/10 of the subjects were randomly selected as the validation set. The area under the receiver operating characteristic (ROC) curve (AUC) was used to evaluate the discrimination of the models, and the DeLong test was used for comparing AUCs between models. The Hosmer-Lemeshow test and calibration curves were used to evaluate the calibration of the models. Decision Curve Analysis (DCA) and Clinical Impact Curves(CIC) were employed to assess the clinical utility of the models. SHapley Additive exPlanations(SHAP) was used for models visualization and interpretation.

**Results:**

Among the 265 participants included in the study, there were 52 males and 26 females in the experimental group of 78 patients, with an average age of 44.5 ± 10.34 years. In the control group of 187 patients, there were 108 males and 79 females, with an average age of 49.8 ± 12.20 years. After differential analysis and LASSO regression screening, WBC, SAA, NLR, MLR, PLR, CAR, and CALLY were selected as the 7 indicators, with no multicollinearity among them. Subsequently, using LR, RF, and SVM for further screening, we established three early diagnostic prediction models for NTM disease in HIV-positive patients. In the training set, The AUC values(AUCs) indicated that the predictive performance of the three models was as follows: Logistic regression model (AUC: 0.850, 95% CI: 0.797–0.903), Random Forest model (AUC: 0.849, 95% CI: 0.797–0.890), and SVM model (AUC: 0.813, 95% CI: 0.750–0.876). The sensitivities were 69.2%, 71.8%, and 76.9%. The specificity values were 89.8%, 85.0%, and 78.6%. The Youden index scores were recorded as 0.590, 0.568, and 0.555. The Positive Likelihood Ratios (LR+) were found to be 6.780, 4.787, and 3.590. The Negative Likelihood Ratios (LR-) were determined to be 0.343, 0.332, and 0.294. The comparison of the AUC values among the three models indicated that there were no statistically significant difference in the predictive efficacy between them. The calibration curves indicated that the predicted probabilities were generally aligned with the observed actual probabilities. With regard to quantitative evaluation, the results of the Hosmer-Lemeshow test were as follows: for the LR model, χ^2^ = 8.078, *p* = 0.426; for the RF model, χ^2^ = 13.081, *p* = 0.1091; and for the SVM model, χ^2^ = 0.620, *p* < 0.001.These findings indicate that both the LR and random forest models exhibit good calibration and accuracy, whereas the SVM model shows poor calibration. Consequently, the SVM model was excluded from further consideration. Consequently, the SVM model was deemed unsuitable and subsequently discarded. The clinical decision curve analysis showed that both the LR and RF models could provide benefits to patients, demonstrating comparable levels of advantage. In the validation set, the ROC curve indicated that the AUC of the LR model was 0.873 (95% CI: 0.782 - 0.961) and that of the RF model was 0.860 (95% CI: 0.768 - 0.952). The calibration curve showed that the predictions of these two models tend to be consistent with the actual values. Hosmer-Lemeshow test: LR model: χ^2^ = 5.111, *p* = 0.746; RF model: χ^2^ = 12.489, *p* = 0.131, indicating that both models have good calibration. The clinical decision curve shows that LR and RF can also bring clinical benefits to patients in the validation set. Both the LR and RF models demonstrated good predictive performance, calibration, and clinical applicability in both the training and validation sets, indicating that these two models have good stability. The Shapley Additive exPlanations (SHAP) were employed to illustrate the decision-making process of the models. The SHAP summary plot revealed that, in the LR model, the feature with the greatest contribution was WBC, while the feature with the least contribution was CAR. In the RF model, the feature with the greatest contribution was PLR, whereas the feature with the least contribution was CALLY.

**Conclusion:**

We have found that WBC, NLR, PLR, CAR, and CALLY can assist in the early identification of HIV-positive patients with NTM disease. Based on established parameters, we have successfully developed two early diagnostic prediction models for HIV-positive patients coexisting with NTM disease. Both models demonstrate strong discrimination, calibration, clinical applicability and stability. In clinical practice, for HIV patients with suspected concurrent mycobacterial infection, after excluding TB, these two models can be used for screening and early identification of HIV concurrent NTM disease.

## 1 Introduction

According to the Joint United Nations Programme on HIV/AIDS, by the end of 2023, a total of 39.9 million people were infected with HIV. In 2023, 1.3 million people had become newly infected with HIV, and 630,000 people died from AIDS-related diseases (1). By the time of the latest reports, nontuberculous mycobacteria (NTM) were classified into approximately 200 species and 13 subspecies, and NTM disease were known to significantly impair patients’ quality of life (2). In clinical practice, for all cases where imaging and clinical manifestations indicated the need to “rule out mycobacterial infection,” our hospital routinely performed rapid tests such as QuantiFERON-TB, Xpert^®^ MTB/RIF (or Xpert^®^ MTB/RIF Ultra), TB-LAMP, and TB-SAT RNA simultaneously. These tests enable prompt confirmation or exclusion of tuberculosis. However, when all the above results were negative but the patient’s symptoms continued to worsen and the imaging showed progression, the most challenging next question for the clinical practice was whether there is an infection of NTM. In a management guideline for HIV-positive patients with NTM disease from South Africa, it was pointed out that the prevalence of NTM in HIV-positive patients were increasing globally, and species identification and drug susceptibility testing were crucial in the diagnosis and treatment of NTM infections (3). It was noted that NTM cultivation is highly demanding, difficult, and time-consuming, with different species requiring distinct culture media and varying incubation periods (4). Moreover, the positive culture rate for NTM disease patients is only about 6.3% (5). Therefore, this study focuses on how to identify NTM early in HIV-suspected mycobacterium-infected patients after TB has been ruled out, in response to the urgent need in real clinical scenarios.

New inflammatory markers are those beyond the traditional indicators such as C-Reactive Protein (CRP), Interleukin 6 (IL6), and Procalcitonin (PCT). They include composite indicators like the Systemic Immune-Inflammation Index (SII), Neutrophil-to-Lymphocyte Ratio (NLR), and Platelet-to-Lymphocyte Ratio (PLR). These markers can provide more information about the inflammatory state and are cost-effective, convenient, and rapid. They have been widely recognized and applied in a variety of diseases (6, 7). However, their value and application in HIV-associated NTM infections have not yet been fully explored. There is an urgent need for in-depth research to fully uncover their potential in the early diagnosis of HIV-associated NTM disease.

For its ability to make automated decisions, handle data efficiently, perform massive computations, and deliver excellent predictive power, machine learning is highly favored. Shapley Additive exPlanations (SHAP) is a method for interpreting the predictions of machine learning models. It is grounded in the concept of Shapley values from game theory, assigning importance values to each feature of the model. By quantifying the impact of each feature on the target variable, SHAP elucidates the prediction process of the model (8).

Based on the clinical necessity for early diagnosis of NTM infections in HIV-positive patients, and taking into account the advantages offered by novel inflammatory indicators—such as economic affordability, easy accessibility, and the high efficiency along with substantial computational capabilities of machine learning in data processing—this paper aims to leverage machine learning to thoroughly investigate the clinical significance of these new inflammatory markers in HIV-positive individuals diagnosed with NTM disease. We will develop an early prediction model for NTM disease specifically tailored for HIV-positive patients and visualize the interpretability of this diagnostic model using the SHAP method.

## 2 Materials and methods

### 2.1 Sample size estimation

Based on the current literature, the prevalence of NTM disease among HIV-positive patients ranges from 11.8% to 47% (9, 10).For this study, the prevalence of NTM disease among HIV-positive patients was determined to be 29.4%. Drawing on a meta-analysis of risk factors for NTM disease (11), an odds ratio (OR) of 4.46 was chosen for a rough estimation of the sample size.

### 2.2 Study participants

**Figure 1:**
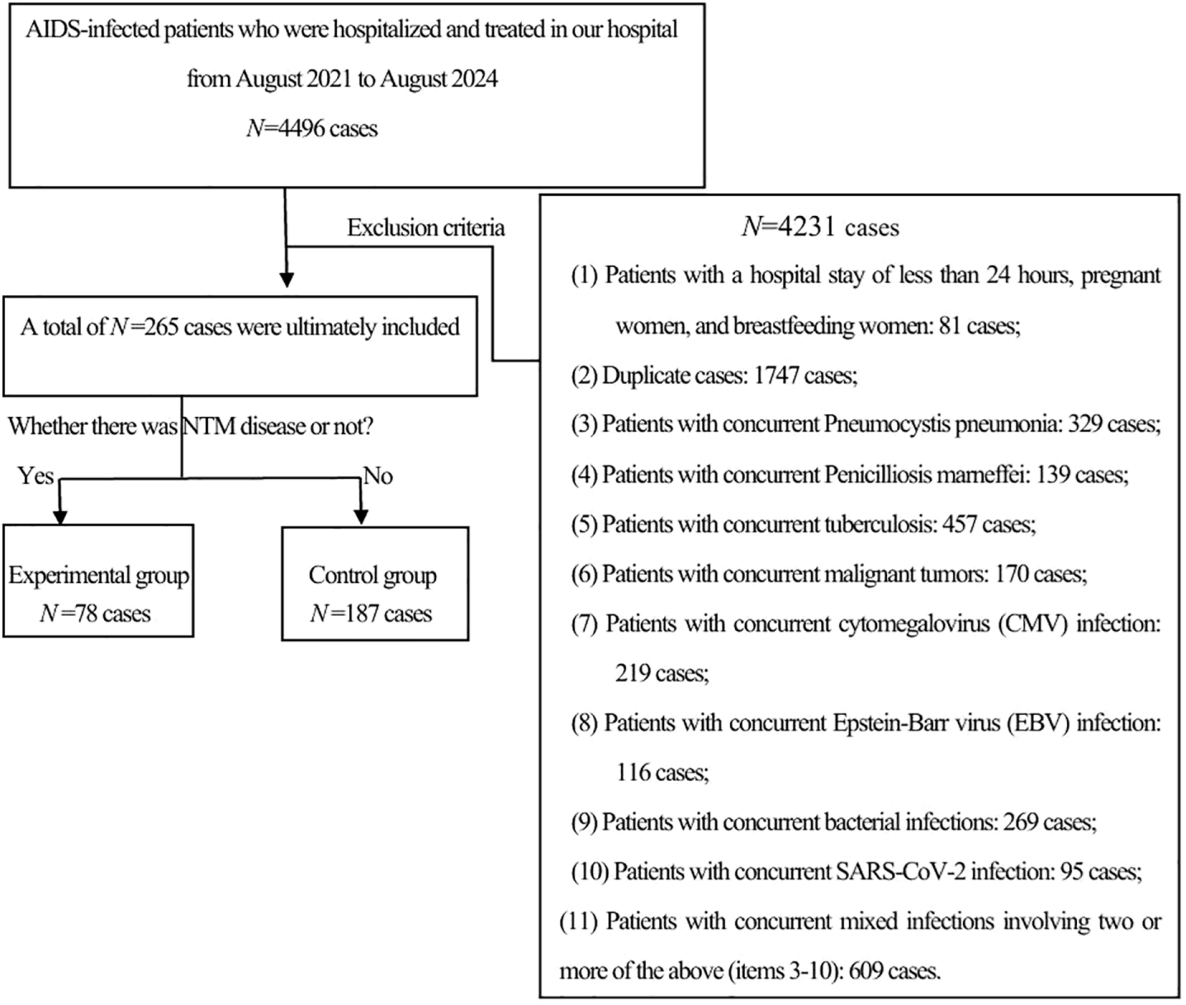
Study participants.

A retrospective analysis was conducted on 4,496 HIV-positive patients who were hospitalized at the Third People’s Hospital of Kunming from August 1, 2021, to August 31, 2024. Inclusion criteria: HIV infection was diagnosed according to the diagnostic criteria specified in the “Chinese HIV/AIDS Diagnosis and Treatment Guidelines (2024)” (12). NTM disease was diagnosed in line with the criteria detailed in the “Guidelines for the Diagnosis and Treatment of Non-tuberculous Mycobacterial Disease (2020)” (13). The exclusion criteria were as follows: (1) patients younger than 18 years old or older than 80 years old; (2) pregnant or breastfeeding women; (3) patients with a hospital stay of less than 24 hours or incomplete data; and (4) patients with other severe infections, severe cardiopulmonary diseases, or malignancies. Based on the inclusion and exclusion criteria, a total of 78 patients with HIV-associated NTM disease were included as the experimental group, and 187 patients with HIV but without NTM disease were included as the control group(See **Figure 1**).

From 2021 to 2024, a total of 4496 HIV patients were hospitalized in our hospital. After excluding 4,231 cases, 265 cases remained. These cases were further divided into an experimental group of 78 cases and a control group of 187 cases based on whether they were complicated with NTM.

### 2.3 Laboratory test

Routine blood tests were performed using the Sysmex XN400 analyzer (Sysmex Corporation, Japan), with 3–5 ml of whole blood collected within 24 hours of admission.

### 2.4 Data collection

We collected data on the gender, age, ethnicity, medical history, and laboratory test results of the study participants: (1) Laboratory data included the following test indicators: white blood cell count (WBC), monocyte count (M), lymphocyte count (L), neutrophil count (N), platelet count (PLT), albumin (ALB), creatinine (CREA), serum amyloid A (SAA), procalcitonin (PCT), C-reactive protein (CRP), and interleukin-6 (IL-6).

(2)Calculation of novel inflammatory markers: see **Table 1** below.

**Table 1:** Calculation of novel inflammatory markers.

Indicator name	Indicator name	Calculation formula
Systemic immune inflammation index	SII	(PLT×N)/L
Neutrophil to lymphocyte ratio	NLR	N/L
Platelet to lymphocyte ratio	PLR	PLT/L
Monocyte to lymphocyte ratio	MLR	M/L
Neutrophil-to-platelet ratio	NPR	N/PLT
Platelet to albumin ratio	PAR	PLT/ALB
CRP to albumin ratio	CAR	CRP/ALB
Albumin to creatinine ratio	ACR	ALB/CREA
Neutrophil to albumin ratio	NAR	N/ALB
CRP to lymphocyte ratio	CLR	CRP/L
Systemic inflammatory response index	SIRI	(N×M)/L
Proinflammatory value	PIV	(N×PLT×M)/L
CALLY index	CALLY	(ALB×L)/CRP
Inflammatory load index	ILI	(CRP×N)/(ALB×L)
White blood cell index	WBI	(N+L)×M

### 2.5 Statistical analysis

Data were analyzed using SPSS 27.0, R 4.4.2, and Python 3.12. Variables with a missing data rate of ≥70% were discarded, and those with a missing rate of <30% were imputed using multiple imputation. Categorical data were expressed as rates and proportions, and intergroup comparisons were performed using the chi-square test. Continuous data that followed a normal distribution were expressed as mean ± standard deviation $\bar{x}$±s and intergroup comparisons were conducted using the t-test. The non-normally distributed data were described as M(Q1, Q3) and compared using a non-parametric test for two independent samples. Variable selection was performed using Least Absolute Shrinkage and Selection Operator regression(LASSO), Logistic regression(LR), Random Forest(RF), and Support Vector Machine - Recursive Feature Elimination (SVM-RFE). LASSO was implemented using the “glmnet” package with the following parameters: alpha = 1, nfolds = 10, and standardize = TRUE. LR was carried out using the “stats” package with the “logit” link function. The RF was built using the “randomForest” package with ntree = 500 and importance = TRUE. SVM-RFE was performed using the “e1071” and “caret” packages with the parameter setting of method = “svmLinear”. To assess multicollinearity among variables, the tolerance and variance inflation factor (VIF) were used. LR, RF, and SVM models were constructed. All the subjects included in the study were assigned to the training set, and 3/10 of the subjects were randomly selected as the validation set. Model evaluation was applied to R packages such as pROC, caret, CalibrationCurves, ResourceSelection, rmda, ggplot2 and et al. The discrimination of the models was evaluated using the area under the receiver operating characteristic (ROC) curve (AUC), and comparisons of AUC between models were conducted using the DeLong test. The calibration of the models was evaluated using the Hosmer-Lemeshow test and calibration curves, while the clinical utility of the models was assessed using decision curve analysis (DCA) and clinical impact curves(CIC). The model was visualized and interpreted using SHAP. The SHAP library was applied, and the summary_plot, dependence_plot, force_plot, decision_plot, and heatmap functions were called. A two-sided test was used, with a significance level of α = 0.05. A p-value of less than 0.05 was considered statistically significant.

## 3 Results

### 3.1 Comparison of baseline characteristics between the experimental group and the control group

Among the 265 study subjects, the analysis of baseline characteristics had shown that: as seen in **Table 2**, there was no statistically significant difference between the experimental and control group in terms of gender, ethnicity, route of HIV infection, marital and reproductive, smoking history, as well as the history of diabetes, hypertension, and alcohol consumption (*p* > 0.05). Only age had statistically significant differences between the two groups (*p* < 0.05). Among them, in the experimental group of 78 patients, there were 52 males and 26 females, with a mean age of 44.5 ± 10.337 years, and 46 patients (59.0%) were smokers; in the control group of 187 patients, there were 108 males and 79 females, with a mean age of 49.8 ± 12.203 years, and 84 patients (44.9%) were smokers.

**Table 2:** Comparison of baseline characteristics of the study subjects.

Variables	Experimental (*n*=78)	Control (*n*=187)	Statistical value	*p*	
Gender(M)	52(66.7%)	108(57.8%)	1.828	0.176
Ethnicity (Han)	72(92.3%)	171(91.4%)	0.429	0.807
Age (Years)	44.5±10.337	49.8±12.203	3.386	0.001^**^
Diabetes(Yes)	5(6.4%)	21(11.2%)	1.445	0.229
Hypertension(Yes)	8(10.3%)	33(17.6%)	2.299	0.129
Smoking(Yes)	45(57.7%)	93(49.7%)	1.397	0.237
Drinking(Yes)	31(39.7%)	65(34.8%)	0.592	0.442
Routes of HIV infection					
Intravenous drug use	4(5.1%)	12(6.4%)		
Sexual transmission	48(61.5%)	101(54.0%)	1.283	0.526
Others	26((33.3%))	74(39.6%)		
Marital status					
Unmarried	20(25.6%)	28(15.0%)		
Married	47(60.3)	127(67.9%)	4.058	0.255
Others	11(14.1%)	32(17.1%)		

The comparison of traditional and novel inflammatory markers between the two groups showed that: as presented in **Table 3**, the traditional inflammatory markers WBC, CRP, SAA, IL-6, and PCT had statistically significant differences between the two groups (*p* < 0.05); among the 15 novel inflammatory markers, SII, NLR, MLR, PLR, CAR, CLR, SIRI, CALLY, and ILI had statistically significant differences between the two groups (*p* < 0.05).

**Table 3:** Comparison of traditional and novel inflammatory markers between the control and experimental group.

Variables	Experimental(*n*=78)	Control (*n*=187)	Statistical value	*p*
WBC	4.59(3.10,5.76)	5.86(4.61,7.07)	-4.395	<0.001^***^
NEUT	3.14(1.82,4.61)	3.39(2.42,4.39)	-1.294	0.196
CRP	23.47(2.91,56)	2.62(0.95,9.43)	-6.033	<0.001^***^
SAA	107(5.88,234.11)	3.7(1.1,16.6)	-6.189	<0.001^***^
IL6	14.45(4.76,47.85)	3.55(1.92,7.12)	-6.366	<0.001^***^
PCT	0.08(0.04,0.27)	0.04(0.02,0.07)	-4.616	<0.001^***^
SII	682.41(428.82,1699.76)	391.97(266.22,636.62)	-5.083	<0.001^***^
NLR	3.99(2.25,10.06)	1.90(1.36,2.92)	-6.674	<0.001^***^
MLR	0.51(0.35,0.92)	0.28(0.20,0.39)	-7.182	<0.001^***^
PLR	286.24(185.56,408.68)	134.40(96.37,171.43)	-8.027	<0.001^***^
NPR	0.02(0.01,0.03)	0.02(0.01,0.02)	-0.591	0.555
PAR	5.97(4.22,7.72)	5.46(4.40,7.03)	-1.002	0.316
CAR	0.96(0.08,2.33)	0.07(0.02,0.26)	-6.083	<0.001^***^
ACR	0.57(0.45,0.77)	0.62(0.53,0.80)	-1.761	0.078
NAR	0.10(0.06,0.16)	0.09(0.06,0.12)	-1.050	0.294
CLR	30.63(3.37,162.60)	1.60(0.52,7.06)	-6.771	<0.001^***^
SIRI	1.40(0.79,3.78)	0.84(0.59,1.50)	-3.890	<0.001^***^
PIV	225.37(103.97,627.28)	180.80(105.34,302.45)	-1.896	0.058
CALLY	1.10(0.21,12.64)	23.96(5.14,78.20)	-7.362	<0.001^***^
ILI	3.47(0.21,24.73)	0.12(0.04,0.63)	-6.574	<0.001^***^
WBI	10.4(7.67,14.23)	11(8.84,13.72)	-0.805	0.421

*p < 0.05, **p < 0.01, ***p < 0.001.

### 3.2 WBC, SAA, NLR, MLR, PLR, CAR, and CALLY were identified as significant factors

**Figure 2:**
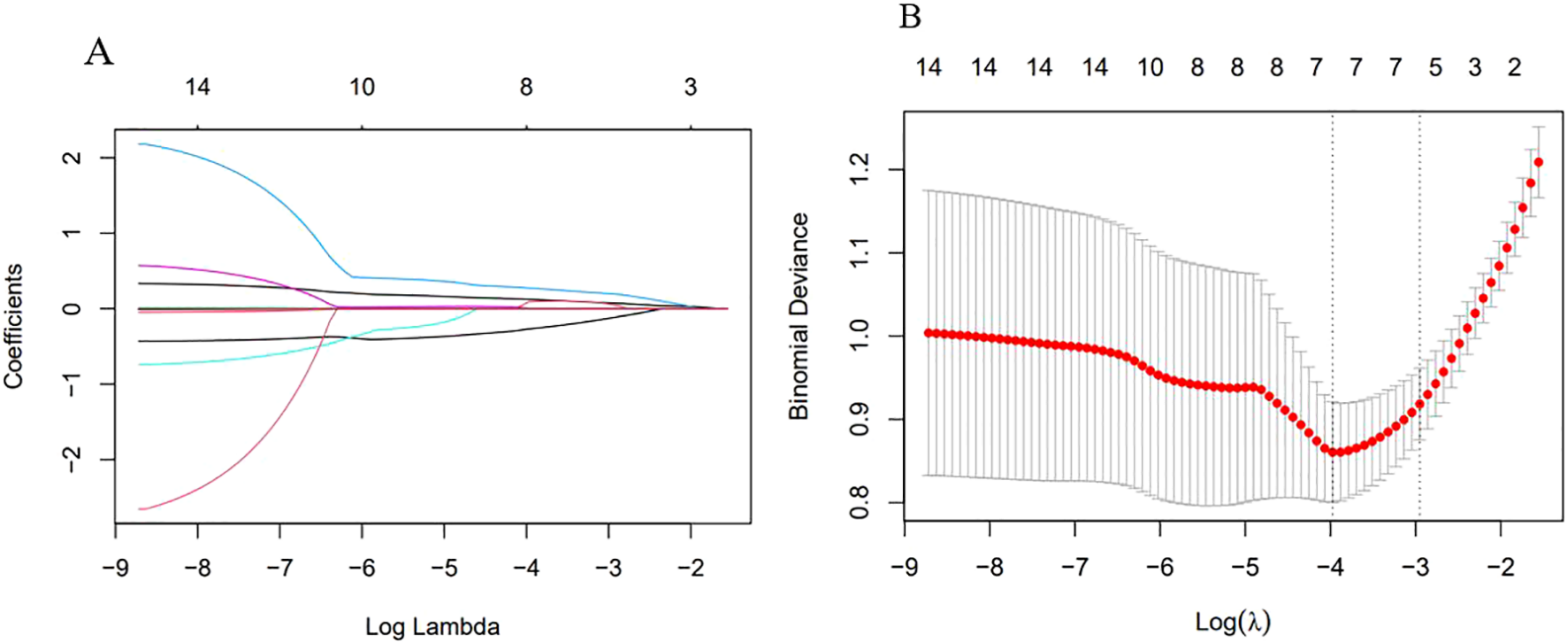
Variable selection by Lasso regression. **(A)** λ and Partial Regression Coefficient Plot. The horizontal axis represented log(λ), the upper axis corresponded to the number of non-zero coefficients; the vertical axis represented the partial regression coefficient values of each variable. It indicated the order in which variables enter the LASSO regression model and the coefficient trajectory as λ decreases. **(B)** Ten-fold Cross-validation Error Curve. The horizontal axis represented log(λ), the upper axis corresponded to the number of non-zero coefficients; the vertical axis represented the mean squared prediction error (MSE) of the model. The left dotted line indicated the log(λ) at the minimum error, retaining 7 variables; the right dotted line indicated the log(λ) under the “1-standard error” criterion, retaining 5 variables.

**Figure 3:**
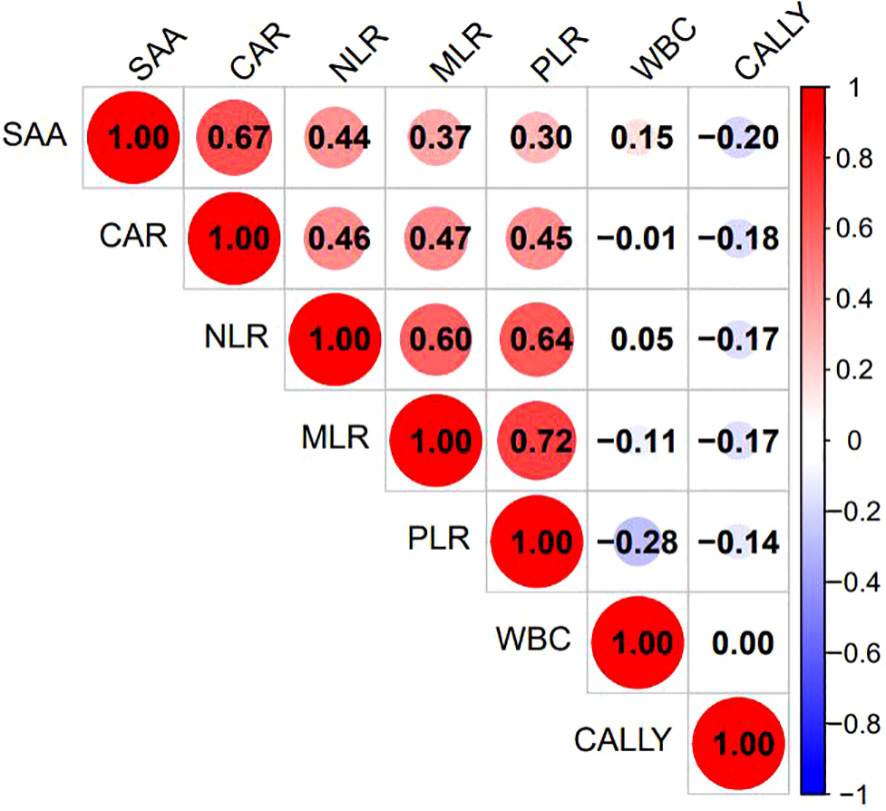
Heatmap of correlations among the 7 selected indicators. The various inflammatory indicators of behavior were listed as corresponding indicators. The figure showed the Pearson correlation coefficient(r), with the color gradient ranging from blue (r = –1) to red (r = +1), and white indicating r=0.

Excluding the indicators NEUT, NPR, PAR, ACR, NAR, PIV, and WBI due to their lack of statistical significance, Lasso regression screening was performed on the remaining 14 inflammatory markers of both new and old types. The methodology is illustrated in **Figure 2A**. Through a process of 10-fold cross-validation, seven indicators—WBC, SAA, NLR, MLR, PLR, CAR, and CALLY—were ultimately selected (as depicted in **Figure 2B**). Subsequently, multicollinearity tests were conducted on these seven indicators. The absolute values of the correlation coefficients (R) between each pair of indicators were all found to be less than 0.8 (as shown in **Figure 3**), and the Variance Inflation Factor (VIF) for each indicator was below 3. These results indicate that there is no multicollinearity present among the selected seven indicators.

### 3.3 Three diagnostic models, specifically LR, RF, and SVM, were developed to forecast the occurrence of HIV in conjunction with NTM

The above-mentioned 7 indicators were included in the binary logistic regression analysis, with the method selected as “Forward: LR.” Ultimately, the variables entered into the equation were shown to be WBC, NLR, PLR, and CAR (as shown in **Table 4**). This indicates that these 4 inflammatory indicators are independent influencing factors for HIV-infected patients with NTM coinfection. Using the 4 indicators as key factors, a LR model was established. The 7 indicators selected by Lasso regression were ranked by importance using random forests, and the top 4 indicators (PLR, CALLY, NLR, WBC) were chosen as key factors to establish a RF model. The 7 indicators were also ranked by importance using SVM-RFE, and the top 4 indicators (PLR, CALLY, NLR, CAR) were selected as key factors to establish an SVM model.

**Table 4:** Analysis of independent influencing factors for HIV coinfected with NTM.

Variables	*β*	*SE*	*Wald χ*2	*p*	*OR*(95% *CI*)
WBC	-0.411	0.111	13.668	<0.001***	0.663(0.533~0.824)
NLR	0.205	0.088	5.408	0.020*	1.228(1.033~1.459)
PLR	0.005	0.002	6.363	0.012*	1.005(1.001~1.009)
CAR	0.512	0.193	7.043	0.008**	1.669(1.143~2.436)

*p < 0.05, **p v 0.01, ***p < 0.001.

### 3.4 Comparison and evaluation of models

#### 3.4.1 The three models—LR, RF, and SVM—demonstrate strong predictive performance in forecasting the occurrence of HIV in conjunction with NTM

**Figure 4:**
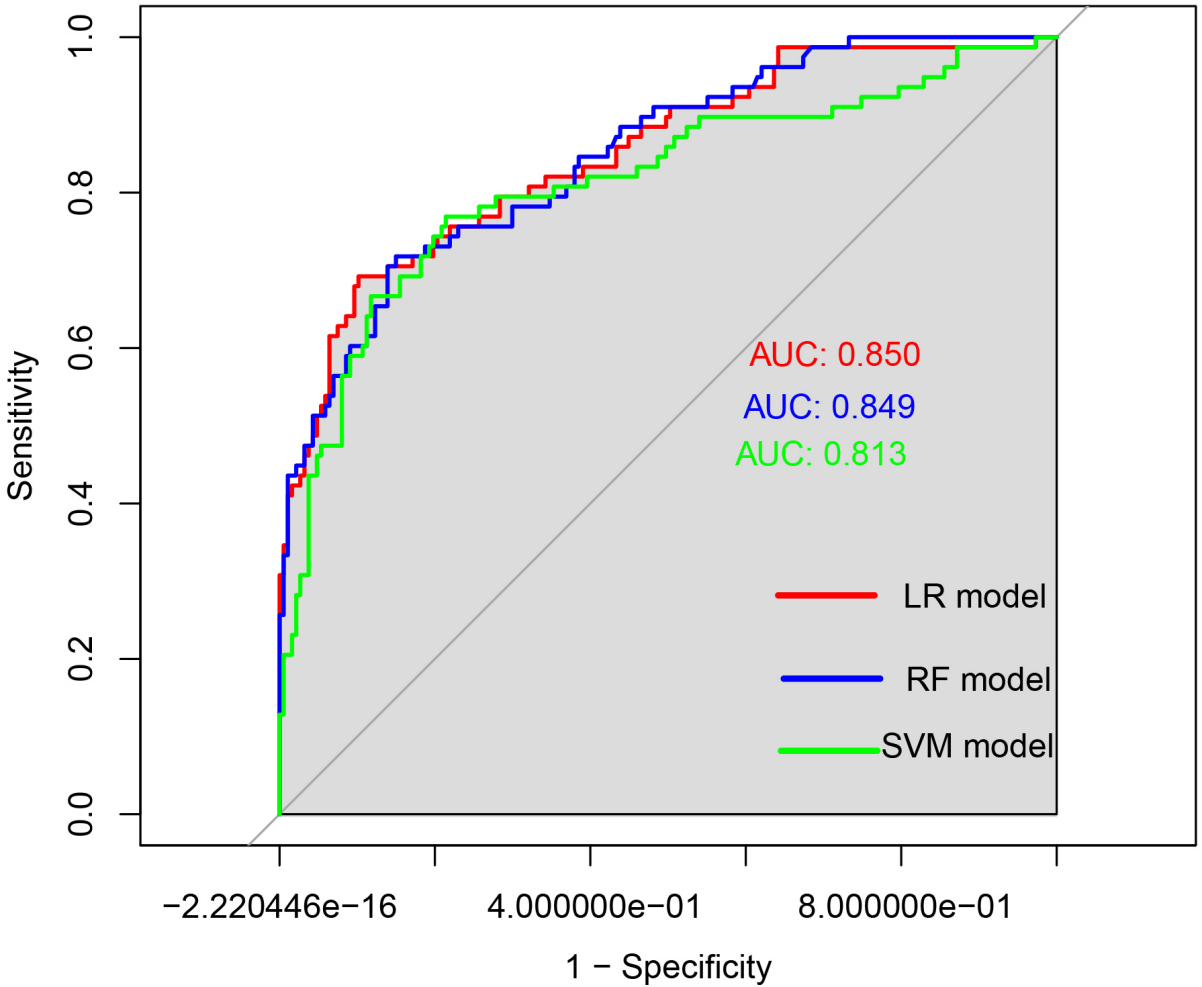
ROC curves of the three models for predicting the risk of HIV complicated with NTM. The x-axis represented 1-specificity, and the y-axis represented sensitivity. The red curve illustrated the ROC curve of the logistic regression model used for predicting HIV in conjunction with NTM disease. The blue curve depicted the ROC curve of the random forest prediction model for forecasting HIV concurrent with NTM disease. Lastly, the green curve represented the ROC curve of the support vector machine model aimed at predicting HIV alongside NTM disease.

The ROC curves were generated, and the areas under the curve (AUC) were compared to assess the predictive efficacy of the LR,RF and SVM models for the patients in the experimental group. The predictive efficacy in descending order was as follows (see **Table 5**, **Figure 4**): the LR model (AUC: 0.850, 95%CI: 0.797 - 0.903), the RF model (AUC: 0.849, 95%CI: 0.797 - 0.890), the SVM model (AUC: 0.813, 95%CI: 0.750 - 0.876). The sensitivity rates were recorded at 69.2%, 71.8% and 76.9%. The specificity rates were noted as 89.8%, 85.0% and 78.6%. The Youden index were 0.590, 0.568 and 0.555. The Positive Likelihood Ratio (LR+) were found to be 6.780, 4.787 and 3.590. The Negative Likelihood Ratio (LR-) were recorded as 0.343, 0.332 and 0.294. These findings indicate that the three models possess a certain predictive value for HIV complicated by NTM.

**Table 5:** ROC curves analysis of the three models for predicting the risk of HIV complicated with NTM.

Models	AUC(95%*CI*)	Optimal cutoff value	Sensitivity	Specificity	Youden’s Index	LR+	LR-	*p*
LR model	0.850(0.797~0.903)	0.630	69.2%	89.8%	0.590	6.780	0.343	<0.001^***^
RFmodel	0.849(0.797~0.890)	0.653	71.8%	85.0%	0.568	4.787	0.332	<0.001^***^
SVM model	0.813(0.750~0.876)	0.244	76.9%	78.6%	0.555	3.590	0.294	<0.001^***^

***p < 0.001.

When the AUCs of the three models were compared (see **Table 6**), it was found that the AUC of the LR model was higher than that of the other two models. However, the differences in AUC among the three models were not statistically significant (*p*> 0.05).

**Table 6:** Comparison of ROC curves for the three models.

Models comparison	ΔAUC	Z	*p*	95%CI
LR model vs RF model	0.004	0.0831	0.9338	0.036~0.039
LR model vs SVM model	0.037	1.6654	0.0958	-0.007~0.081
RF model vs SVM model	0.035	1.2860	0.1984	-0.019~0.089

#### 3.4.2 The LR and RF models demonstrated strong calibration in predicting the concurrent occurrence of HIV and NTM. However, the performance of the SVM model was found to be unsatisfactory

**Figure 5:**
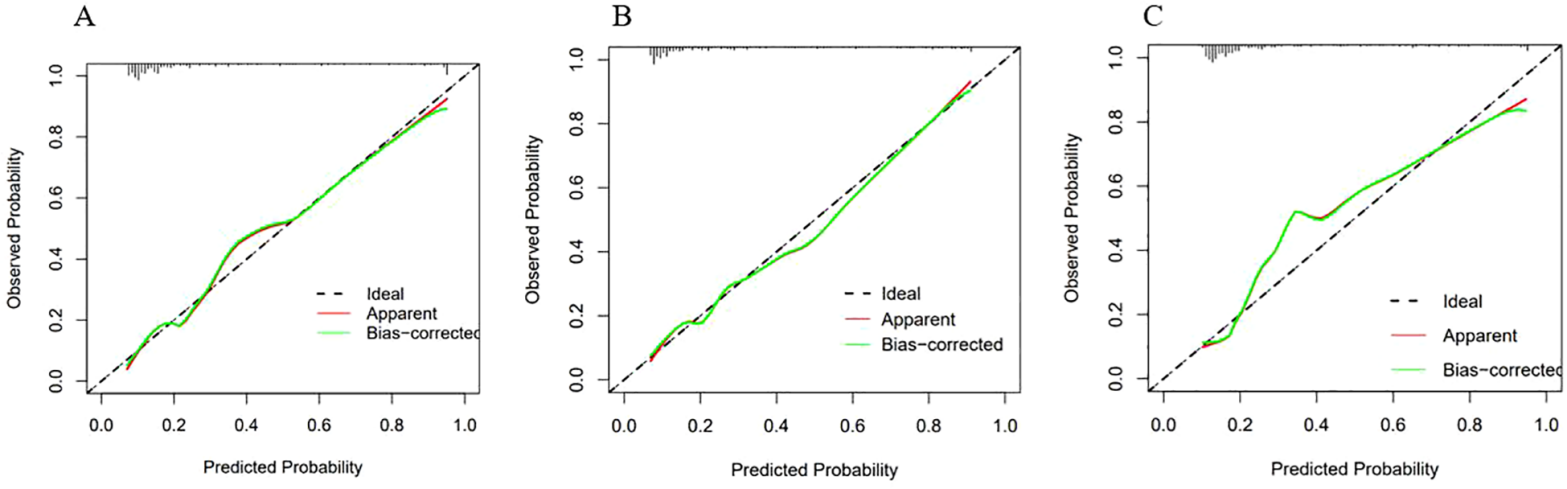
Calibration curves of the three models. The X-axis represented the predicted probability, and the Y-axis represented the actual observed occurrence probability. The closer the curve was to the 45° dotted line (Ideal line), the more consistent the predicted probability was with the actual incidence rate. **(A)** Calibration curve of the Logistic regression model; **(B)** Calibration curve of the Random Forest model; **(C)** Calibration curve of the SVM model.

Calibration curves were generated for each of the three models, as illustrated in **Figure 5**. They were observed that the predicted and actual values of the LR and RF models exhibit a strong alignment, closely following the diagonal line. However, the calibration curve of the SVM model exhibited a deviation from the diagonal line.

The Hosmer-Lemeshow test was used to quantify the differences between the predicted and actual values. The test results were as follows: for LR model, χ^2^ = 8.078, *p* = 0.426, indicating that there was no statistically significant difference between the predicted and actual values of this model; for RF model, χ^2^ = 13.081, *p* = 0.1091, indicating that there was no statistically significant difference between the predicted and actual values of this model.

Considering both the calibration curves and the Hosmer-Lemeshow test, the LR and RF models demonstrated satisfactory calibration. These two models demonstrated a high level of accuracy in distinguishing the presence of NTM disease.

For the SVM model,χ^2^ = 0.620, *p* < 0.001, indicating that there was a statistically significant difference between the predicted and actual values of this model. This finding suggests that the SVM model exhibited poor accuracy in distinguishing whether NTM disease was present. Therefore, this model was discarded.

#### 3.4.3 Both the LR and RF models demonstrated considerable potential for application in clinical settings

**Figure 6:**
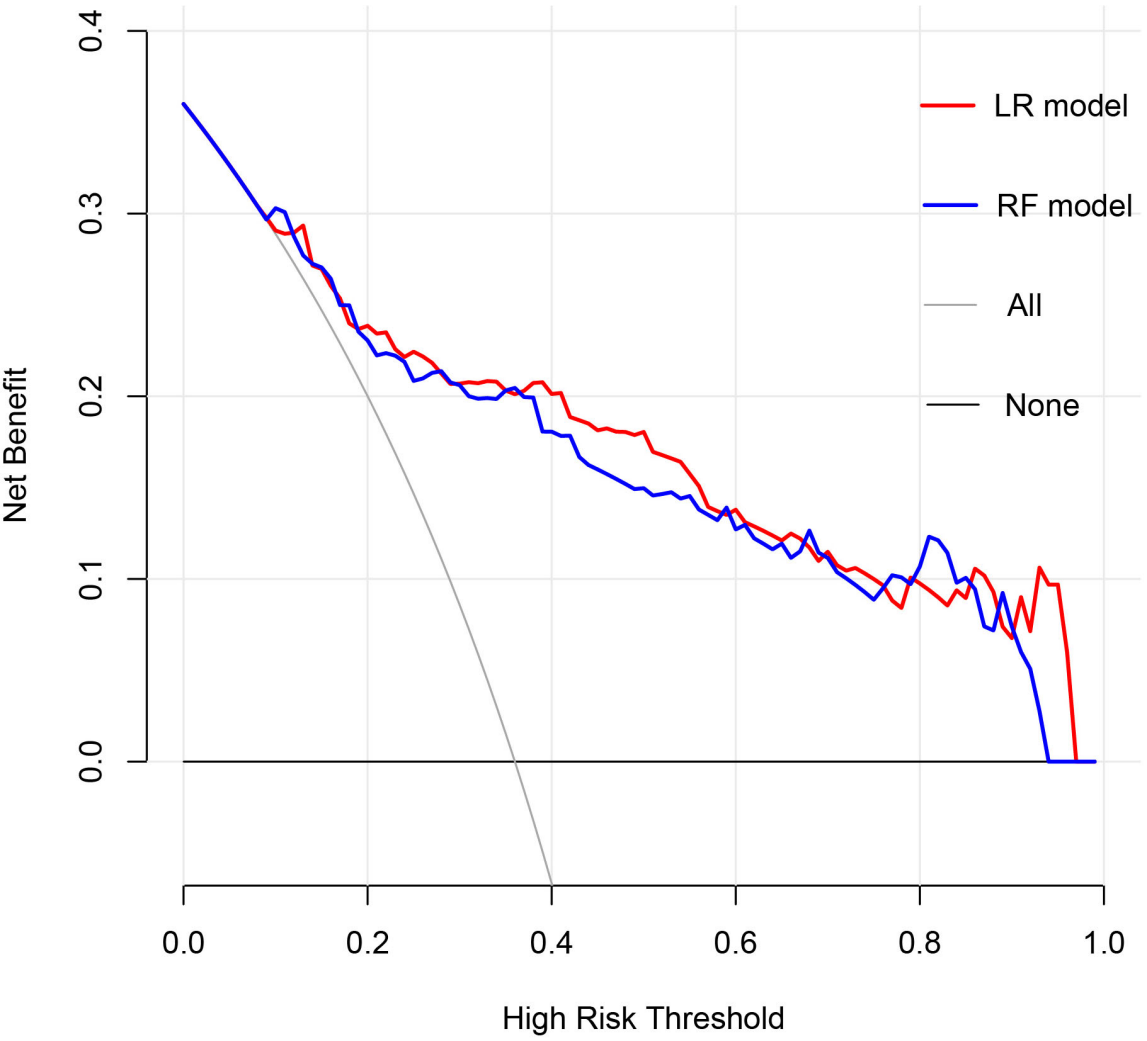
Clinical Decision Curve. The X-axis represented the high risk threshold, and the Y-axis represented the net benefit. The curve showed that at different risk thresholds, the clinical net benefit of the models were higher than that of the “intervene all” or “no intervention” strategies. The red curve represented the clinical decision curve of the logistic regression model; the blue curve represented the clinical decision curve of the random forest model. They suggested that the two models have good clinical practicability.

The clinical utility of the models was evaluated through the use of clinical decision curves, as illustrated in **Figure 6**. The decision curves of the two models were predominantly situated above the two extreme lines. With the optimal cutoff values of 0.630 and 0.653 for the two models as the threshold probabilities, the net benefit rates of the two models were greater than zero and higher than the net benefit rates under the two extreme scenarios. The net benefit rates of the two models were close to each other. This indicated that both models could benefit patients in a similar manner and had good clinical applicability.

#### 3.4.4 In the validation set, both the LR and RF models exhibited outstanding predictive performance, calibration accuracy, and clinical applicability

**Figure 7:**
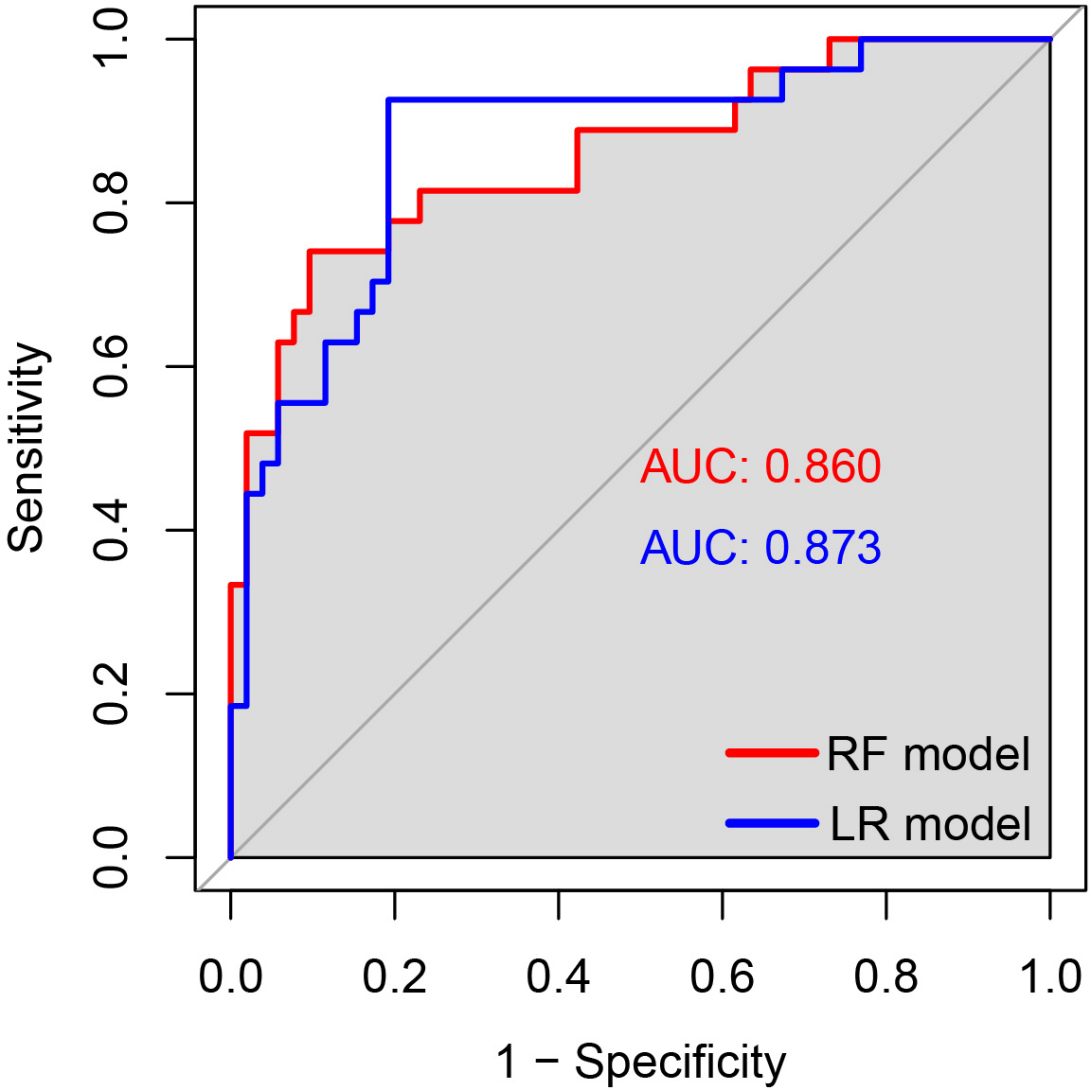
The ROC curves of the LR and RF models for predicting the risk of HIV combined with NTM in the validation set. The x-axis represented 1-specificity, and the y-axis represented sensitivity. The red curve illustrated the ROC curve of the random forest model for predicting HIV concurrent with NTM disease in the validation set, while the blue curve represented the ROC curve of the logistic regression model for predicting HIV concurrent with NTM disease in the same validation set.

**Figure 8:**
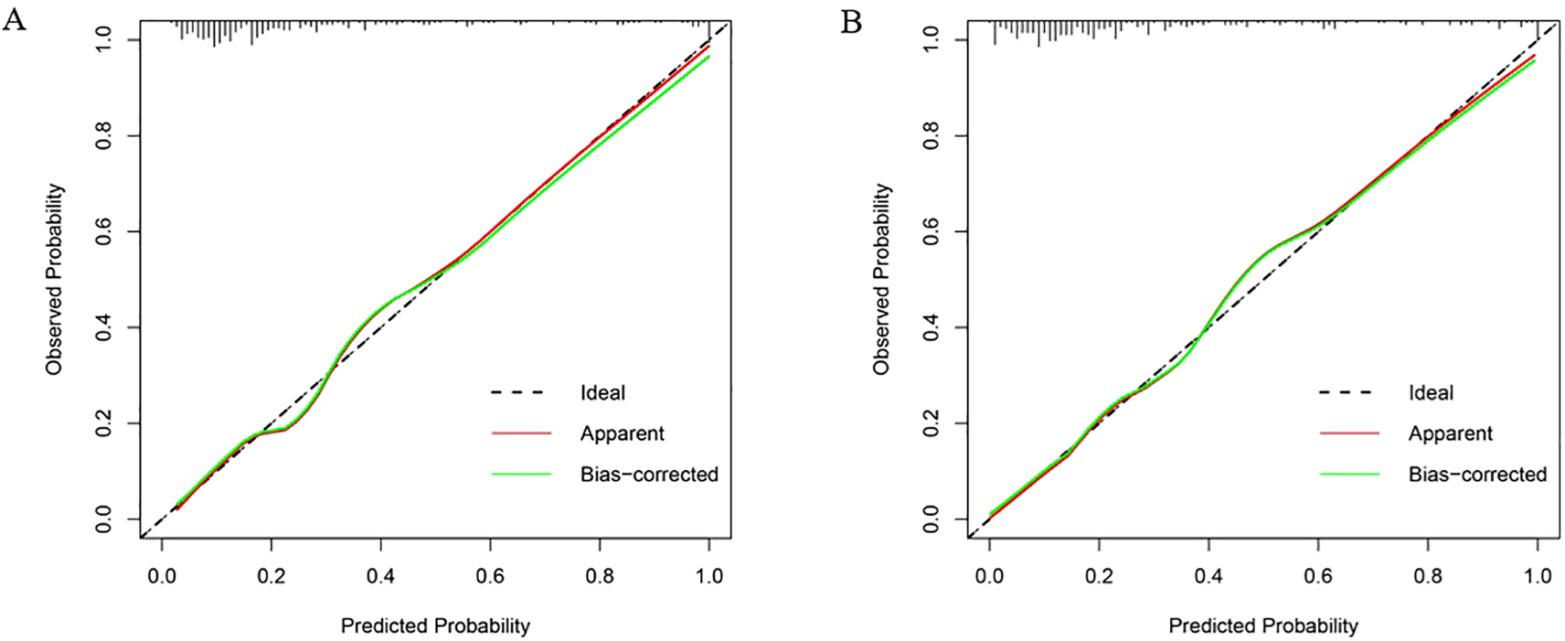
Calibration curves of LR and RF models in the validation set. **(A)** The X-axis represented the predicted probability, and the Y-axis represented the actual observed occurrence probability. The closer the curve was to the 45° dotted line (Ideal line), the more consistent the predicted probability was with the actual incidence rate. Calibration curve of the logistic regression model in the validation set; **(B)** Calibration curve of the random forest model in the validation set.

**Figure 9:**
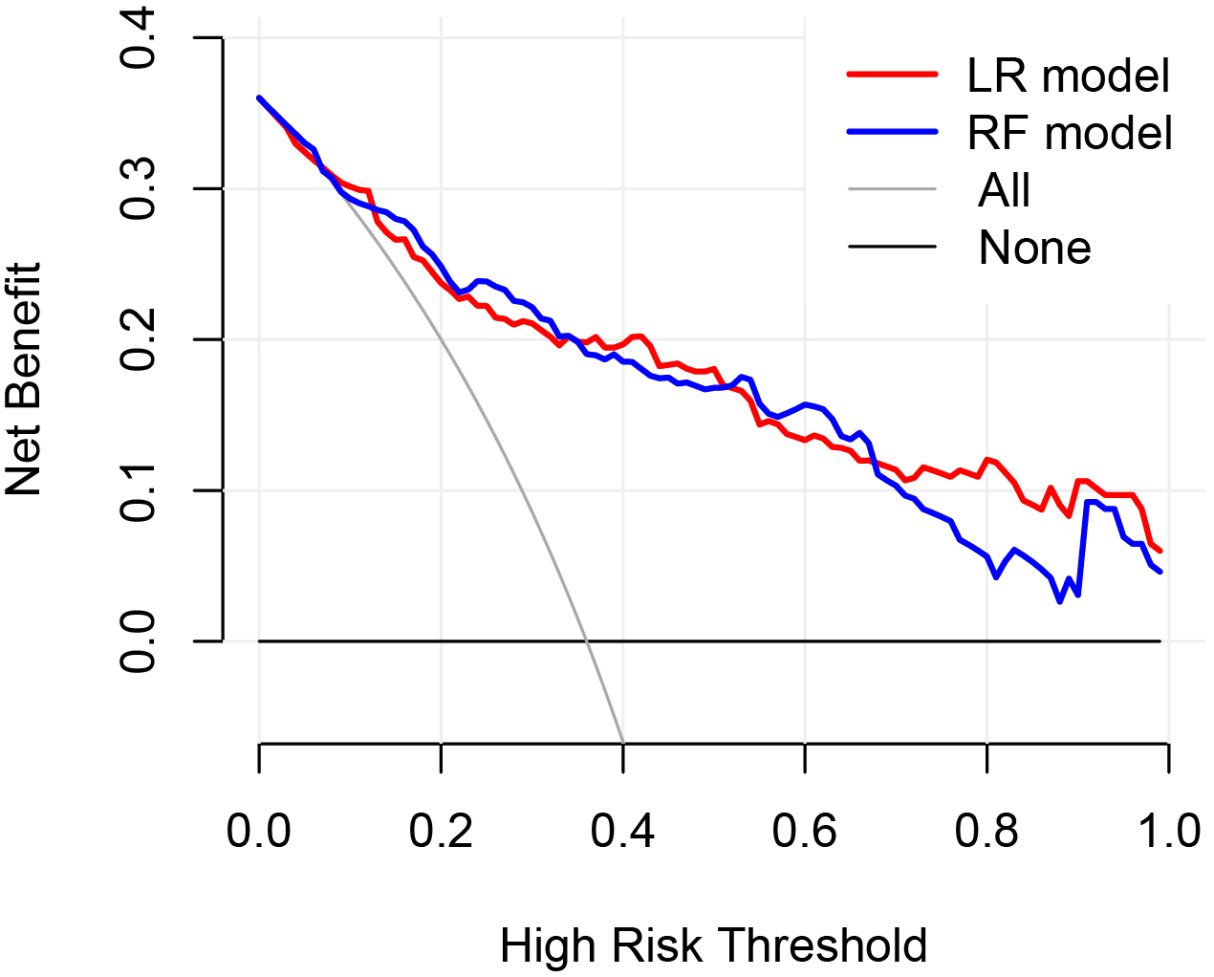
Clinical decision curves of the LR and RF models in the validation set. The X-axis represented the high risk threshold, and the Y-axis represented the net benefit. The curve showed that at different risk thresholds, the clinical net benefit of the models were higher than that of the “intervene all” or “no intervention” strategies. The red curve represented the clinical decision curve of the logistic regression model in the validation set; the blue one was that of the random forest model in the validation set. This indicated that in the validation set, both models also have good clinical practicability.

**Figure 10:**
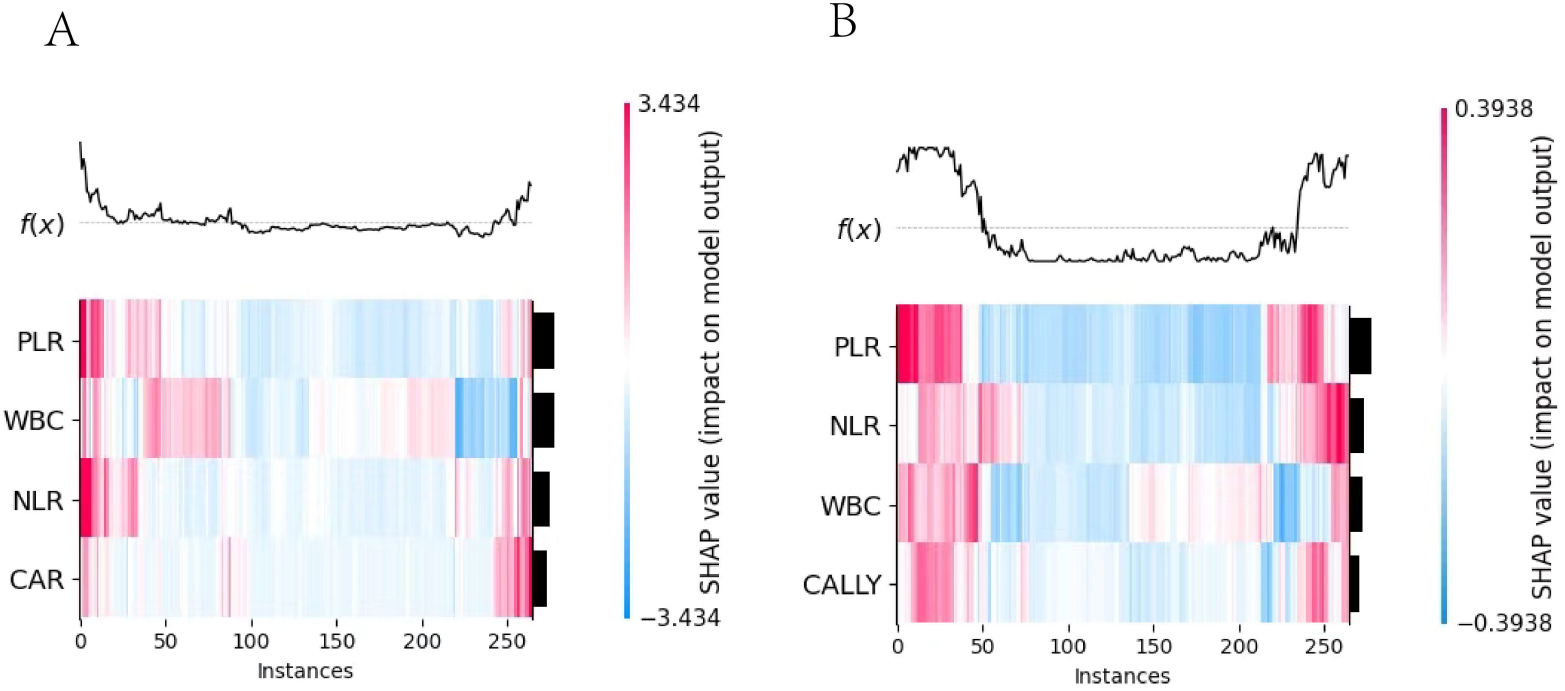
SHAP heatmap. 1. **(A)** the Logistic regression model heatmap; **(B)** the Random Forest model heatmap. 2. The x-axis represented the number of instances, the y-axis represented the impact of features on instances, and the color indicated the extent and direction of the impact. (red for positive, blue for negative).

In the validation set, first, the AUCs were plotted, and the predictive performance of the models in sequence (**Figure 7**) was as follows: the LR model (AUC: 0.873, 95% CI: 0.782-0.961) and the RF model (AUC: 0.860, 95% CI: 0.768-0.952). The AUCs of both models were greater than 0.8, indicating that these two models also had good predictive performance in the validation set. Secondly, the calibration curves for the LR and RF models were plotted using the validation set, as illustrated in **Figure 8**, indicating that the predictions of these two models tend to be consistent with the actual values. The Hosmer-Lemeshow test showed that for the LR model: χ^2^ = 5.111, *p* = 0.746, and for the RF model: χ^2^ = 12.489, *p* = 0.131, indicating that there was no statistically significant difference between the predicted values and the actual observed values for these two models. The results of the comprehensive calibration curve and Hosmer-Lemeshow test indicated that the LR and RF models also have good calibration in the validation set. Finally, the clinical decision curves of the LR and RF models were plotted, as shown in **Figure 9**: The decision curves of both models were mostly above the two extreme lines, indicating that LR and RF also demonstrated the potential to benefit patients in the validation set. Both the LR and RF models demonstrated good predictive performance, calibration, and clinical utility in both the training and validation sets, indicating that these two models have good stability, as shown in **Figure 10**.

### 3.5 SHapley Additive exPlanations explains the models

#### 3.5.1 Visual analysis and interpretation of multi-feature contribution based on SHAP heatmap

The important feature rankings were shown on the left y-axis, with features sorted by their influence from greatest to smallest. Their visualizations were presented on the right y-axis. In the image, the depth of color indicates the magnitude of the SHAP values, which reflect the impact of the feature values on the model. The deeper the color, the larger the absolute value of the SHAP value and greater the influence on the model. The top part of the image visualizes the model’s prediction results based on these values.

#### 3.5.2 Feature contributions: WBC dominated LR, CAR contributed least; PLR Led RF, CALLY trailed

**Figure 11:**
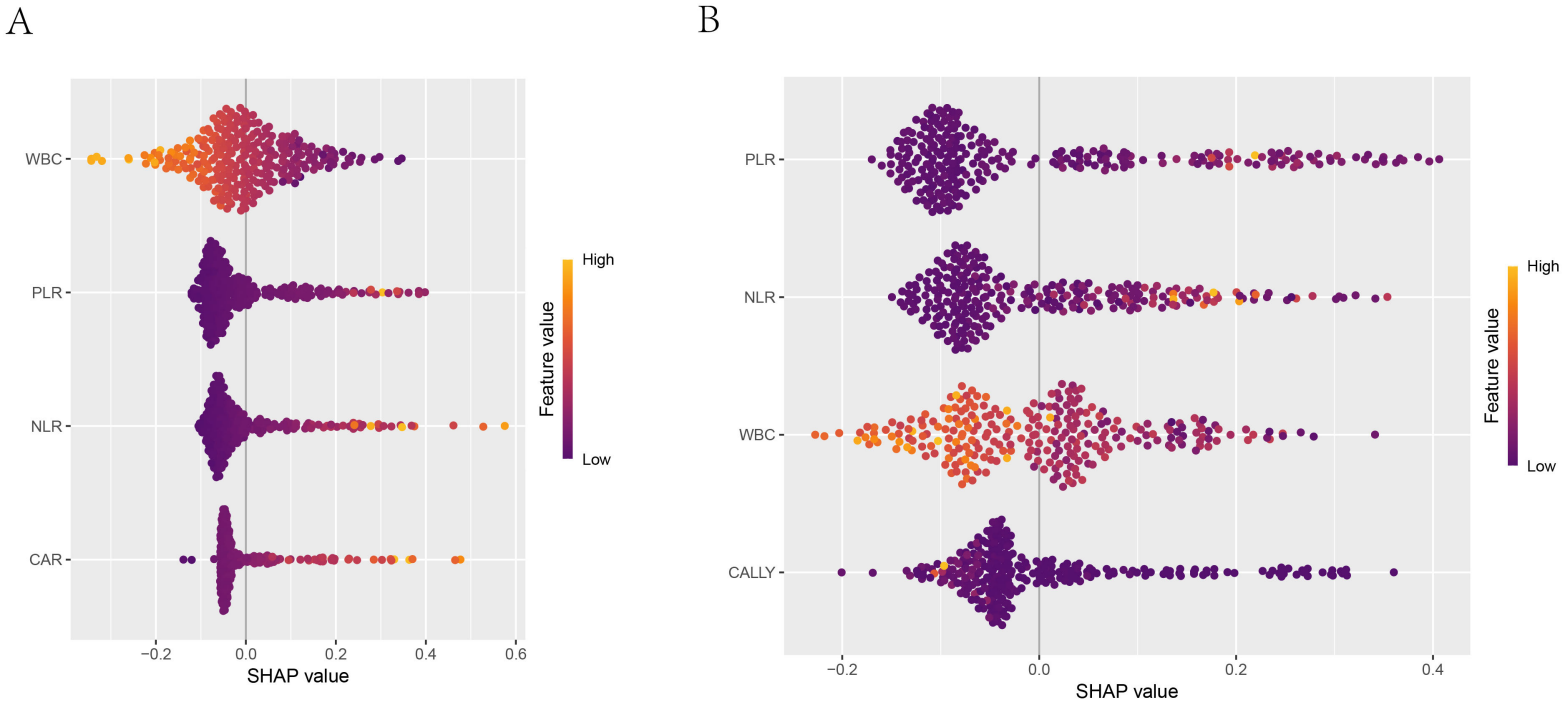
SHAP summary plot. 1. **(A)** Summary plot of the logistic regression model; **(B)** Summary plot of the random forest model. 2. The x-axis shown the SHAP values of each feature, indicating the impact of each feature on the prediction outcome. The y-axis shown the importance ranking of the features. The color indicated the direction of the feature’s impact on the model’s prediction, with orange-yellow representing positive impact and blue-purple representing negative impact.

The SHAP summary plot of the LR model (as shown in **Figure 11A**) revealed that among the four indicators used for modeling, WBC was the most contributive to the model, with its different levels exerting significantly varying impacts on the model’s prediction outcomes. In contrast, CAR was the least contributive indicator. The SHAP summary plot of the RF model (as shown in **Figure 11B**) indicated that among the four indicators used for modeling, PLR was the most contributive to the model, with a predominantly negative impact on the model’s prediction outcomes across the vast majority of its levels. In contrast, CALLY was the least contributive. A comparison of the two plots revealed that the contributions of the same indicators varied across different models.

#### 3.5.3 The correlation between feature variables and target variables

**Figure 12:**
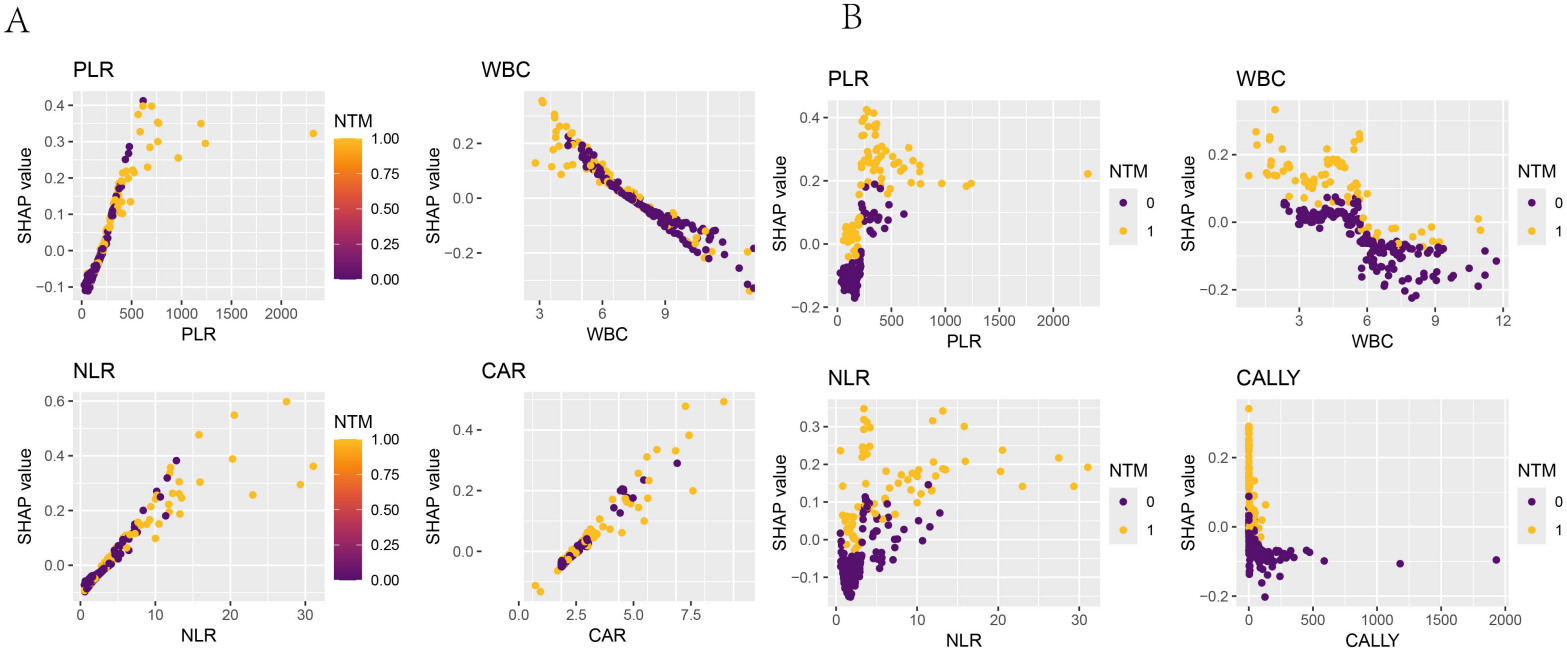
SHAP dependence plot. The x-axis represented the variable values, and the y-axis represented the SHAP values of the model. The color indicated the model output variable, with dark blue representing HIV-positive patients without concurrent NTM and yellow representing HIV-positive patients with concurrent NTM. **(A)** SHAP dependence plots of PLR, WBC, NLR, and CAR in the logistic-regression model with respect to “whether concurrent NTM”. **(B)** SHAP dependence plots of PLR, WBC, NLR, and CALLY in the random-forest model with respect to “whether concurrent NTM”.

The SHAP dependence plots have illustrated the relationships between the features and the target variable. **Figure 12A** have shown the relationships of the four features used to build the Logistic regression model with the target variable (whether NTM disease was present), while **Figure 12B** have depicted the relationships of the four features used to build the RF model with the target variable. For instance, PLR was positively correlated with concurrent NTM in both models.

#### 3.5.4 The decision-making process of the research subjects in the model

**Figure 13:**
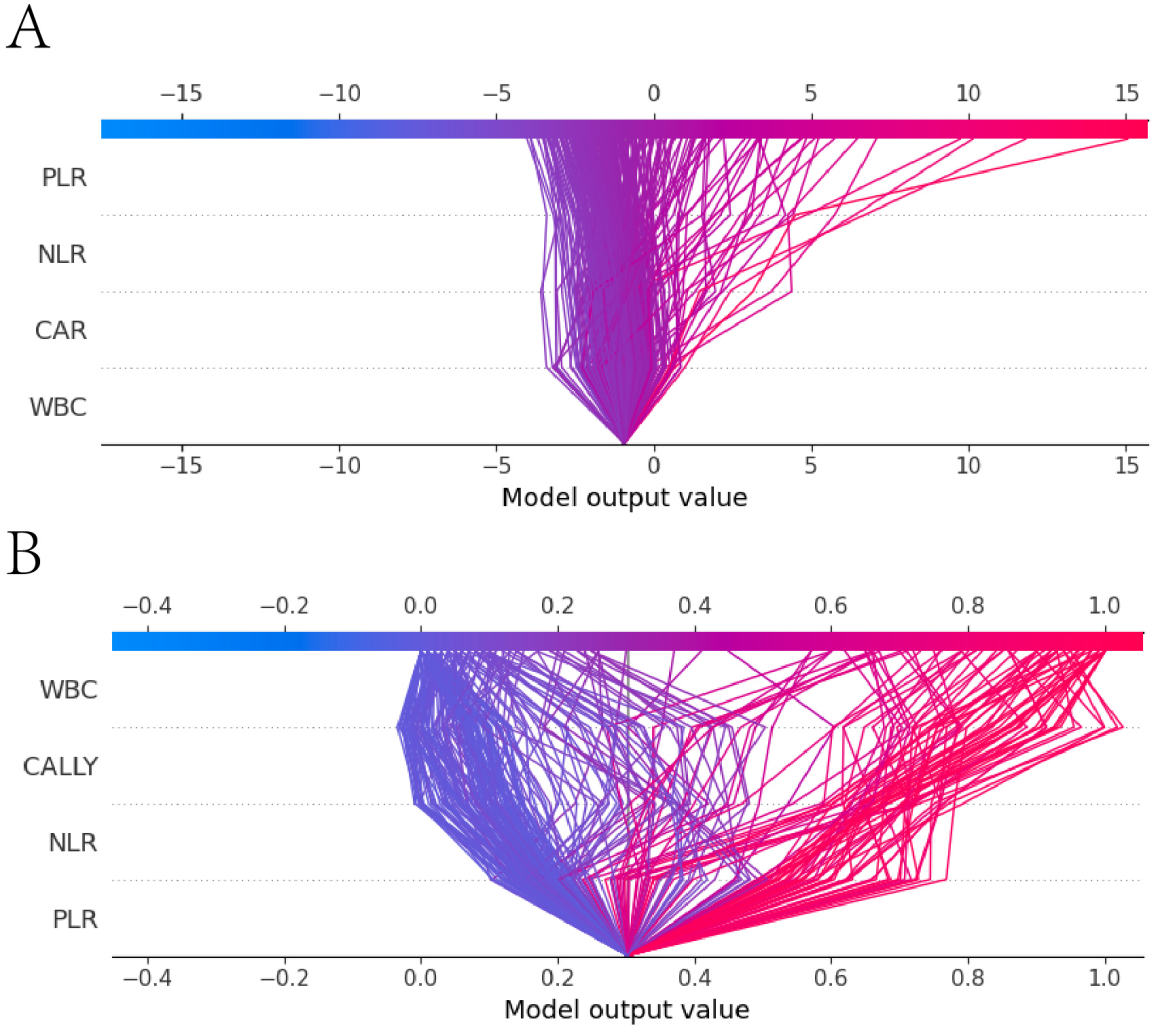
SHAP decision plot. **(A)** the SHAP decision plot for the Logistic regression model; **(B)** the SHAP decision plot for the Random Forest model. The x-axis represented the feature values, with the first horizontal line indicating the starting point of the prediction and the second horizontal line indicating the endpoint of the prediction. The color denoted the direction of the prediction. The lines between the two horizontal lines shown the prediction path. The y-axis represented the features involved in the model’s decision - making process.

The SHAP decision plot illustrated how each feature contributed to the model’s output results. **Figure 13A** shown the decision - making process of 265 subjects in the LR model, while **Figure 13B** shown the decision-making process of 265 patients in the RF model.

#### 3.5.5 The LR and RF models demonstrated accurate predictions when discriminating between random and external validation singletons

**Figure 14:**
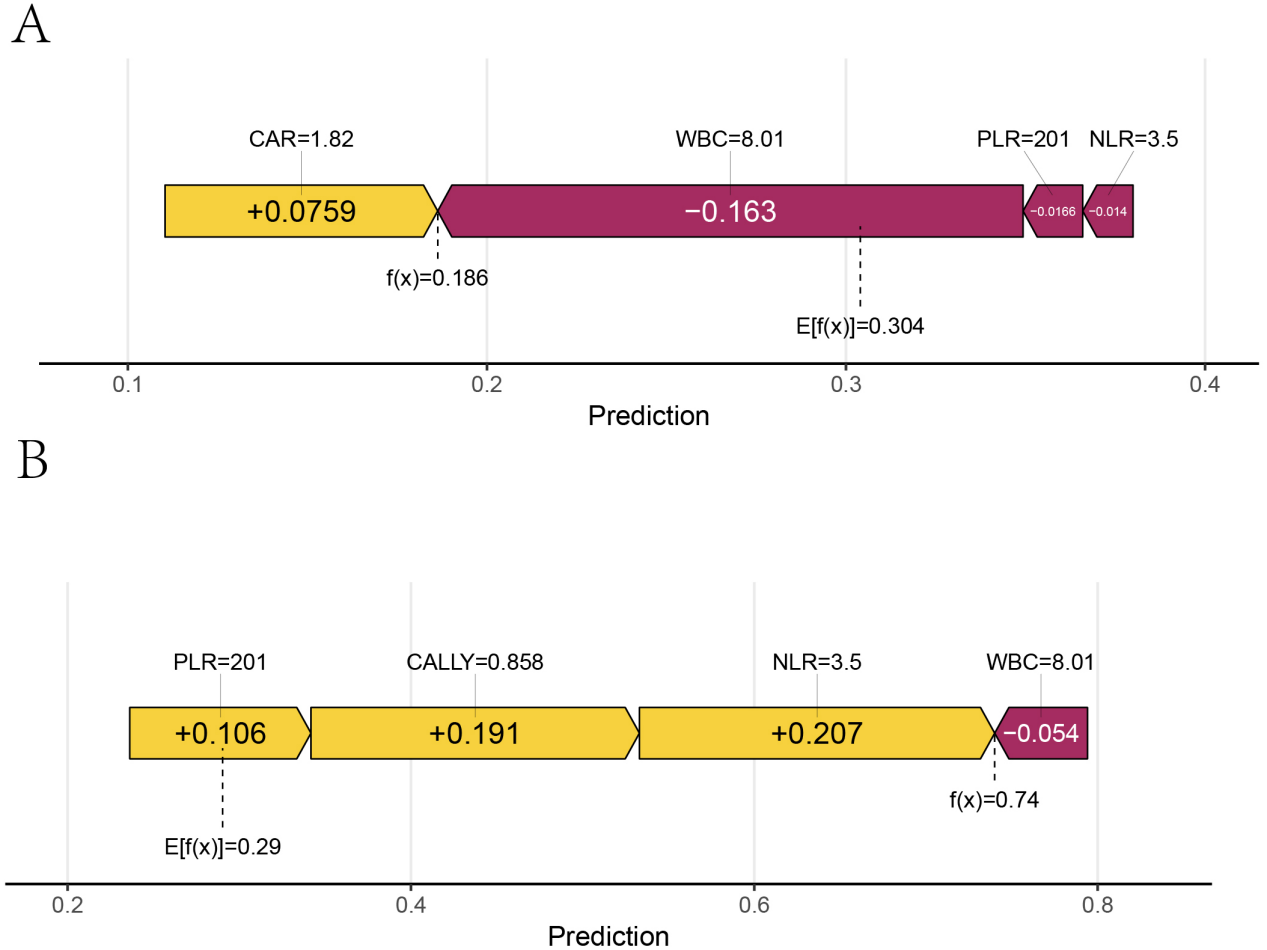
Single sample force plot. 1. Figures **(A, B)** were force plots of the same patient based on different models. A was based on the Logistic regression model, and B was based on the Random Forest model. 2. E[f(x)] represented the baseline value of the model, and f(x) represented the final prediction value. The length of each feature’s bar indicated its contribution to the prediction value, and the color indicated the direction of influence, with orange-yellow representing positive impact and dark red representing negative impact.

**Figure 15:**
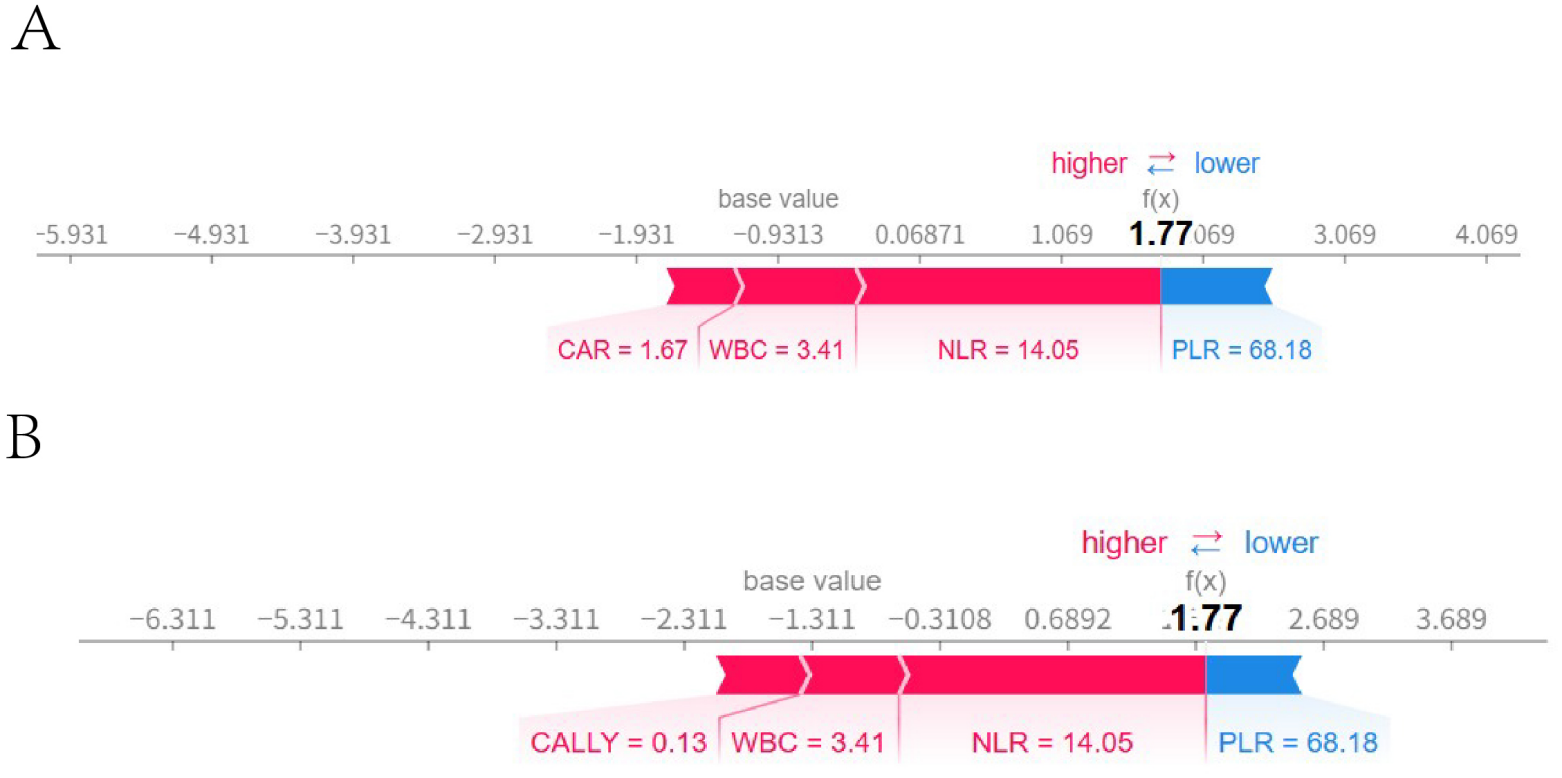
External validation single sample ROC curve. Figures **(A, B)** were force diagrams of the same patient based on different models (The patient’s clinical information: PLR: 68.18, NLR: 14.05, CAR: 1.67, CALLY: 0.13, NTM: 1). A was based on the logistic regression model, while Bwas based on the random forest model. The base value represented the model’s baseline value, and f(x) represented the final predicted value. The length of each feature bar indicates its contribution to the predicted value, and the color indicated the direction of the influence, with blue representing a positive influence and red representing a negative influence.

A sample was randomly selected, with the following feature values: PLR: 101, WBC: 6.22, NLR: 3.17, CAR: 1.33, CALLY: 1, NTM: 0 (indicating that the patient did not have NTM). SHAP single - sample force plots were drawn for the two models, as shown in **Figure 14**. The single sample force plot of the LR model, as shown in **Figure 14A**, indicates that the baseline value (E[f(x)]) of this sample in the Logistic regression model is 0.291, which is the starting point of this force plot. The most influential indicator is PLR, with CAR positively contributing 0.0422, PLR negatively contributing 0.0821, WBC negatively contributing 0.0523, and NLR negatively contributing 0.0248. The final predicted value f(x) is 0.174. The predicted risk is 1/(1 + exp(-f(x)), that is, the predicted risk is 0.524, which is less than the cutoff value of 0.630 (see **Table 5**), and is judged as no concurrent NTM, consistent with the actual result. In the RF model of this sample, as shown in **Figure 14B**, it is indicated that E[f(x)] = 0.302, f(x) = 0.098, and the measured risk is 0.525, which is less than the optimal cut-off value of 0.653 (see **Table 5**). Since f(x) is less than 0.653, it is judged as not concurrent with NTM, which is consistent with the actual result. The information of the only patient diagnosed with HIV concurrent with NTM disease in our hospital from September 1st to 30th, 2024 will be selected for further verification. The patient’s clinical information: PLR: 68.18, NLR: 14.05, CAR: 1.67, CALLY: 0.13, NTM: 1. The single sample force diagrams corresponding to the two models were drawn separately, as shown in **Figure 15**. The single sample force diagram of the LR model is shown in **Figure 15A**, with the final predicted value f(x) = 1.77, and the corresponding predicted risk is 0.855, which is greater than the optimal value of 0.630, indicating a concurrent NTM. In the RF prediction model, this sample is shown in **Figure 15B**, with the final predicted value f(x) = 1.77, and the corresponding predicted risk is 0.855, which is greater than the optimal value of 0.653, indicating HIV concurrent NTM disease. The prediction results of the two models are consistent with the actual situation.

## 4 Discussion

In this study, the new and old inflammatory indicators had different values in the early diagnosis of HIV-associated NTM disease. WBC, NLR, PLR, CAR, and CALLY had assisted in identifying individuals with HIV-associated NTM. The LR models and RF models based on these indicators were established, which have shown good diagnostic value for the early identification of HIV-associated NTM.

In this study found that the WBC level in individuals with HIV-associated NTM disease was significantly lower than those without concurrent NTM disease (*p* < 0.001).This might be due to the direct impact of the HIV on WBC, especially the reduction of CD4+ T lymphocytes, which leads to the suppression or deficiency of the immune system and subsequently results in a decrease in WBC.

The reduction in WBC cannot effectively identify and eliminate infections, leading to the occurrence and progression of NTM infections (14, 15). NLR is considered to reflect the balance between innate and adaptive immune responses and is widely used in clinical practice to predict the severity, prognosis and risk of death in patients with various diseases, such as chronic obstructive pulmonary disease (COPD) (16), acute pancreatitis (17), malignant tumors (18), and HPV (19). In infectious diseases, similar value also exists. Shangguan X et al. discovered (20) that NLR levels are associated with the survival of sepsis patients and can be used to predict the 30-day mortality rate of sepsis patients and identify those at high risk of death. Some scholars have also found that NLR is associated with poor prognosis of tuberculosis, and high levels of NLR can significantly increase the adverse outcomes of tuberculosis treatment (21). The NLR has also been linked to mortality risk in people living with HIV (22), and it demonstrates strong discriminatory ability for identifying concurrent human papillomavirus infection in this population, achieving an AUC of 0.882 (19).In this study, the NLR level in HIV patients co-infected with NTM was significantly higher than that in the control group, and the difference was statistically significant (*p* < 0.05). In the multivariate analysis, NLR was an independent risk factor for NTM in HIV patients. Therefore, NLR can be used as an auxiliary indicator to help clinicians assess the inflammatory status of HIV-infected individuals and assist in the early prediction of NTM infection risk, thereby guiding treatment decisions.

PLR reflects the balance between platelets and lymphocytes in patients. Studies have shown that PLR can serve as an assessment indicator for disease progression in HIV patients and can also predict HIV-related complications. It is particularly valuable in predicting the risk of cardiovascular and cerebrovascular diseases such as HIV-associated hypertension (23) and stroke (24), as well as the risk of malignant tumors such as rectal cancer (25), liver cancer (26), and lymphoma (27). Furthermore, elevated PLR levels may inform treatment decisions, influencing whether to initiate or modify antiretroviral therapy (28, 29). In this study, the PLR level in the HIV patients with NTM disease was higher than that in the control group. Multivariate Logistic regression analysis showed that PLR was an independent risk factor for NTM disease in HIV patients. Moreover, the LR model and RF model established with PLR as a participant both had good diagnostic value. The increase in PLR may reflect the intensification of the inflammatory state in HIV-infected individuals, and is related to the severity of NTM infection and immune activation (30). Therefore, PLR not only facilitates the assessment of complications in HIV-infected individuals but also serves as a useful indicator for predicting the risk of HIV concurrent NTM.

The C-reactive protein to albumin ratio (CAR), serving as a comprehensive index for assessing inflammation and nutritional status, has demonstrated significant application value across various clinical domains. Elevated levels of CAR were associated with adverse outcomes in a range of diseases, including cancer (31), acute heart failure (32), chronic obstructive pulmonary disease (33), and sepsis, among others. Chenciner L et al. noted (34) that the CAR serves as a potential inflammatory marker for adult HIV infection, with CAR levels exhibiting an increase during the early stages of HIV infection. Research has demonstrated that C-reactive protein (CRP) levels are elevated (35), while albumin (ALB) levels are significantly reduced (36) in HIV patients who also have concurrent NTM disease. Theoretically, it follows that CAR levels should likewise show a significant increase.

This is consistent with our results. CAR was significantly elevated in the HIV co-infected NTM disease group compared to the control group. Multivariate analysis also showed that a high level of CAR was an independent risk factor for concurrent NTM disease. Our research shows that CAR can effectively distinguish whether HIV patients are complicated with NTM disease. Clinicians should enhance their understanding and clinical application of CAR.

The CALLY index, derived from CRP, ALB, and lymphocyte count, provides a comprehensive reflection of the body’s immune, nutritional, and inflammatory status. Studies have shown that the CALLY index is independently associated with gastrointestinal malignancies (37) and is linearly negatively correlated with the mortality rate of cancer patients (38). It has also been found that there was a significant negative correlation between the CALLY index and the all-cause mortality and cardiovascular mortality among the elderly (39). Moreover, a cross-sectional study showed that the CALLY index was correlated with the disease activity of rheumatoid arthritis (40). However, there is no relevant research on this index in HIV-associated NTM disease. In our study, the CALLY index was significantly lower in the HIV-associated NTM disease group than in the control group. In the SVM-RFE and random forest feature importance rankings, the CALLY index was among the top 4. Moreover, the random forest model established with its involvement could predict whether HIV-positive patients had NTM disease relatively well.

In recent years, machine learning has rapidly emerged in clinical research, particularly in the field of HIV, where significant progress was made in the construction and application of related diagnostic or predictive models. Saldana CS et al. (41) developed a machine learning model for predicting the incidence of HIV. Among the 85,224 patients included in the study, the model demonstrated high predictive performance for 2.37% of the newly diagnosed cases. Lin B et al. systematically compared seven machine learning algorithms and ultimately constructed an HIV infection risk prediction model for men who have sex with men (MSM) based on XGBoost, with an area under the curve (AUC) of 0.777 (95% CI: 0.639 - 0.915) (42). Machine learning models have also demonstrated excellent performance in predicting HIV complications and related aspects. Zhang H et al. discovered through machine learning that the levels of inflammatory markers such as IL-2, IL-4, IL-6, IL-10, TNF-α and IFN-γ were significantly elevated in HIV-positive patients with tuberculosis. Moreover, the support vector machine (SVM) model constructed based on these markers demonstrated good predictive potential (43). Zhang H et al. utilized machine learning techniques and found that among HIV-positive patients, those with tuberculosis exhibited significantly elevated levels of inflammatory markers including IL-2, IL-4, IL-6, IL-10, TNF-α, and IFN-γ. Furthermore, the support vector machine (SVM) model established based on these markers demonstrated promising predictive potential (44). In addition, machine learning technology is also applied in the treatment of NTM disease. A research study utilized deep learning techniques to predict the 3-year, 5-year, and 10-year mortality rates of patients diagnosed with Nontuberculous Mycobacteria Pulmonary Disease (NTM-PD) based on chest X-ray images. In the validation set, the area under the curve (AUC) for the model was recorded at 0.865, 0.942, and 0.865 for each respective time interval. Furthermore, when clinical information was incorporated into the model, its predictive performance demonstrated significant enhancement (45).This study established three prediction models for HIV-associated NTM disease based on LR model, RF, and SVM. After evaluating the discrimination, calibration, and clinical applicability of these three models, we successfully developed two prediction models for HIV-associated NTM disease using Logistic regression and random forest. The two models can provide diagnostic clues for the early identification of HIV-positive patients without NTM disease. Moreover, our study also employed SHAP to interpret the models. SHAP is a model interpretation method based on game theory, which aims to enhance the interpretability of models. By calculating the contribution of each feature to the model’s prediction (Shapley value), it provides both local and global explanations (46). In this study, SHAP was used to interpret the importance of each indicator and the decision-making process of the two models, visualize the prediction process, and facilitate understanding and application.

Our study also has certain limitations. First, In the comparison of baseline data, the age of patients in the HIV co-infected with NTM disease group (44.5±10.337years) was significantly younger than that in the control group (49.8±12.203 years), and the difference was statistically significant. However, the 2020 version of the “Diagnosis and Treatment Guidelines for Nontuberculous Mycobacterial Disease” has already pointed out that increasing age is a risk factor for concurrent NTM disease, so the two groups are also comparable. Secondly, due to the relatively small number of patients with HIV concurrent with NTM, in order to ensure the scientific nature of the sample size, we randomly selected 3/10 of the study subjects as the validation set. Although this can support the robustness of the model prediction and increase confidence in the model prediction, strictly speaking, its value is not as high as that of external validation. To address this deficiency, we are also actively seeking remedial measures. We eventually established two diagnostic models. One can be randomly selected for prediction, and the other for validation. Thirdly, in our research design, the control group excluded tuberculosis patients, which simplified the clinical practice diagnostic issues. The differential diagnosis of HIV concurrent NTM and tuberculosis will be the focus of our future research. Finally, our study a single-center retrospective study, and it remained to be validated by enlarging the sample size and conducting multicenter prospective cohort studies.

## 5 Conclusion

We found that WBC, NLR, PLR, CAR, and CALLY can assist in the early identification of HIV-positive patients with NTM disease. Moreover, utilizing these indicators, we have successfully developed two diagnostic models for the early diagnosis of HIV-related NTM disease, specifically LR and RF. Both models have good discrimination, calibration, clinical applicability and stability. and can be used for screening and early identification of HIV- positive patients with NTM disease. In clinical practice, for HIV patients with suspected concurrent mycobacterial infection, after excluding TB, these two models can be used for screening and early identification of HIV concurrent NTM disease.

## Data Availability

The original contributions presented in the study are included in the article/Supplementary Material. Further inquiries can be directed to the corresponding authors.
